# Editorial: Mechanical thrombectomy and development of new devices: emerging trends in rescue strategies for failed mechanical thrombectomy

**DOI:** 10.3389/fneur.2023.1255953

**Published:** 2023-10-04

**Authors:** Ichiro Yuki, Juyu Chueh

**Affiliations:** ^1^University of California (UC) Irvine Medical Center, Irvine, CA, United States; ^2^School of Medicine, Tufts University, Boston, MA, United States

**Keywords:** mechanical thrombectomy (MT), acute ischemic stroke (AIS), intracranial atherosclerotic disease (ICAD), failed thrombectomy, endovascular treatment (EVT)

In 2015, a series of randomized trials led to the widespread acceptance of mechanical thrombectomy (MT) as a treatment for patients with acute ischemic stroke. Particularly, MT for large vessel occlusion (LVO) under specific pre-treatment conditions is now considered the standard of care (1).

Meanwhile, clinical investigations have shifted their focus toward expanding treatment indications by exploring the effectiveness of MT in cases with longer treatment time windows, lower pre-treatment ASPECT scores, distal branch occlusions, and posterior circulations. LVO associated with intracranial atherosclerotic disease (ICAD) is one of the actively discussed conditions. Reports indicate that ICAD accounts for 6% (2) to 29.6% (3) of ischemic strokes, with varying prevalence among ethnic backgrounds, being more common in Asian, African-American, and Hispanic populations.

The challenges in treating ICAD-related LVO are 2-fold. Firstly, diagnosing ICAD based on the initial imaging is often technically impossible due to the lack of contrast filling in the target lesion. As a result, interventionalists need to make a decision once partial recanalization of the target lesion is achieved although differentiating between ICAD-related occlusion and occlusion caused by hard clot or arterial dissection can also be challenging. Secondly, ICAD-related occlusions are known to be associated with a higher rate of post-treatment re-occlusion (4). Therefore, the selection of rescue therapies such as Percutaneous Transluminal Angioplasty (PTA), PTA with stenting (PTAS), and/or antiplatelet therapy (Glycoprotein IIB/IIIA inhibitor) plays a crucial role in maximizing treatment efficacy while minimizing post-procedure complications such as symptomatic intracerebral hemorrhage (sICH).

Numerous non-controlled studies have been conducted to address these issues; however, the optimal timing to shift from MT to PTA/PTAS during the procedure remains unclear. The number of thrombectomy attempts made can provide more convincing evidence of unsuccessful reperfusion/underlying ICAD, but it also raises concerns about intimal damage due to endothelial denudation, vessel perforations or stretching/torsions.

In this Research Topic of Frontiers of Neurology, Deng et al. conducted a retrospective subgroup analysis of the Angel-ACT registry to evaluate the efficacy and safety of PTAS for ICAD-related acute LVO. Of the 1,793 patients enrolled in the Angel-ACT group, 475 patients who met the inclusion criteria were included in the study. The patients were divided into three groups based on treatment methods: (1) Early Rescue Therapy Group: Patients underwent PTA/PTAS after one or no MT attempt, (2) No Rescue Therapy Group: Patients treated only with MT, and (3) Late Rescue Therapy Group: Patients underwent PTA/PTAS after two or more MT attempts.

After propensity score matching, the Early Rescue Therapy group showed better functional outcomes (mRS 0–1) at 90 days compared to the No Rescue Therapy group [adjusted odds ratio (aOR), 0.55, *p* = 0.01] or Late Rescue Therapy group (aOR 0.39, *p* = 0.01). There was no difference in the risk of symptomatic intracranial hemorrhage between the groups.

The authors concluded that once ICAD-related LVO is suspected, early decision-making to perform rescue therapy improves the efficacy of treatment without increasing the risk of post-procedural complications. The relatively poor clinical outcome in the Late Rescue Therapy group, which underwent MT attempts of twice or greater before transitioning to PTA/PTAS, was accounted for by (1) the lower reperfusion rate that can lead to prolonged procedure time and (2) more intimal damage causing vasospasm and intraluminal thrombosis.

This article provides a valuable contribution to the field of neuro-interventional practice by addressing another predicament that interventionalists have to face from time to time. The study provided another evidence that early decision making of shifting the procedure from simple MT to the rescue therapy improves the treatment outcomes of patient with ICAD-related-LVO.

The results above is also consistent with a recently performed large-scale study, the SAINT (Stenting and Angioplasty in Neurothrombectomy) study, which is a multicenter retrospective study evaluating the efficacy of rescue intracranial stenting for failed thrombectomy (2).

In our Research Topic, there is another article that delves into the same subject. Authored by Cai et al., the article is titled “*Rescue intracranial stenting for acute ischemic stroke after mechanical thrombectomy failure: a systematic review, meta-analysis, and trial sequential analysis*.” The authors conducted a meta-analysis and trial sequential analysis of 15 clinical studies (1,595 patients) evaluating the efficacy and safety of rescue stenting for the failed MT. Compared to non-stenting approaches, rescue stenting was associated with better modified Rankin Scale (mRS) scores (0–2), a lower 90-day mortality rate, without increasing the risk of symptomatic intracranial hemorrhage. The trial sequential analysis also confirmed sufficient sample size and statistical power of the meta-analysis concerning mRS scores. Authors concluded that the study supported the use of rescue stenting as an effective and safe treatment for patients with acute ischemic stroke after a failed MT.

As we witness the growing body of positive clinical data regarding the effectiveness of rescue therapy for IACD-related-LVO, it is logical to consider a randomized clinical trial (RCT) as the subsequent phase to gain more clarity on the treatment's clinical advantages. Nevertheless, it is important to exercise caution due to the historical track record of PTA/PTAS for “symptomatic ICAD”, which has been discouraging (5, 6). Recently, another RCT, the CASSIS trial, also failed to show the benefit of PTAS for the treatment of symptomatic severe ICAD (7). Needless to say the “ICAD-related-LVO” and “symptomatic ICAD” are totally different condition. Nevertheless, the occurrence of post-treatment stroke events or deaths within a 1-year timeframe, which range from 8.5 to 19.7% (5, 8), cannot be ignored, and it emphasizes the urgent requirement for new technological advancements or peri-procedural therapies to enhance the safety of the procedure.

The overall efficacy of rescue therapy for failed thrombectomy cases has been improving over the past several years, partially due to the improvement of the peri-procedural antiplatelet therapy. For instance, an increasing number of studies have reported the benefits of utilizing intra-arterial (IA) injection of short-acting IIb/IIIa inhibitors, such as Tirofiban, as a rescue treatment for failed thrombectomy (9). Furthermore, post treatment protocols of antiplatelet therapy have been changing. Interventionalists are now screening patients more frequently using CYP2C19 genetic testing or platelet aggregometry to rule out potential clopidogrel non-responders and proactively using the new-generation antiplatelet agents, such as ticagrelor or prasugrel, which are fast-acting agents with more consistent efficacy compared to the first-generation thienopyridine, clopidogrel. Given that the majority of RCTs in the past were designed to use clopidogrel for post-dual antiplatelet therapy, there is hope that future RCTs may be expected to have better efficacy and safety in the treated arm.

Currently, there are ongoing developments for the ICAD treatment with the introduction of new-generation endovascular stents specifically designed for this condition, including drug-eluting stent systems. Encouraging results have emerged from several clinical studies conducted in China (10). On the other hand, the lack of an appropriate animal model that accurately simulates ICAD poses challenges in conducting preclinical evaluations for these innovative devices. Therefore, there is a pressing need to establish ICAD animal models that effectively replicate post-treatment thromboembolism and in-stent stenosis. By doing so, we can expedite the progress of new device development aimed at treating patients with treatment resistant LVO.

## Author contributions

IY: Conceptualization, Data curation, Formal analysis, Investigation, Methodology, Validation, Writing—original draft, Writing—review and editing. JC: Conceptualization, Investigation, Methodology, Supervision, Validation, Writing—review and editing.
